# Predicting Strain-Specific Metabolic Capabilities in the Genus *Pseudomonas* with a Flux-to-AI Approach Reveals Hidden Cell Envelope Properties

**DOI:** 10.34133/csbj.0190

**Published:** 2026-08-13

**Authors:** Carlos Focil-Espinosa, Christopher Dalldorf, Diego Martinez, Alejandro Zepeda, Cristal Zuniga

**Affiliations:** ^1^ Department of Biology, San Diego State University, San Diego, CA 92182, USA.; ^2^ Facultad de Ingeniería Química, Universidad Autónoma de Yucatán, Campus de Ciencias Exactas e Ingenierías, Mérida 97203, Yucatán, Mexico.; ^3^ Departamento de Posgrado e Investigación, Tecnológico Nacional de México/I. T. Mérida, Mérida, Yucatán, Mexico.; ^4^ DOE Great Lakes Bioenergy Research Center, San Diego State University, San Diego, CA 92182, USA.

## Abstract

Bacteria from the *Pseudomonas* genus are omnipresent in air, soil, and water. They have been widely studied for their broad metabolic versatility and their presence in epidemiological chains, bioproduction, bioremediation, and disease processes. Each year, more genomic sequences are reported in databases and repositories. However, the relationship between genomic variability in *Pseudomonas* strains and the diversity of their metabolic capabilities across environmentally relevant phenotypes remains unknown. Additionally, predictive tools for analyzing different strains within a systematic framework are limited. Here, we reconstructed genome-scale metabolic models (GEMs) of 44 *Pseudomonas* strains from various environments and investigated their capabilities to metabolize different carbon sources and metabolic intermediaries. By systematically testing the substrate utilization of the models, we demonstrate how GEM-predicted capabilities can differentiate between strains and that high metabolic versatility is associated with the predicted ability of the strains to remove toxic compounds while maintaining core functionalities. Hundreds of model simulations were used as input to a machine-learning classification schema, resulting in the identification of metabolic capabilities that better differentiate species. Interestingly, transcription and expression models supported these findings by showing that the metabolic capabilities identified by the Flux-to-AI framework are associated with differences in proteome allocation across strains, including pathways involved in amino acid and carbohydrate metabolism, ultimately revealing strain-specific resource allocation strategies linked to cell-envelope-associated functions.

## Graphical Abstract

**Figure:**
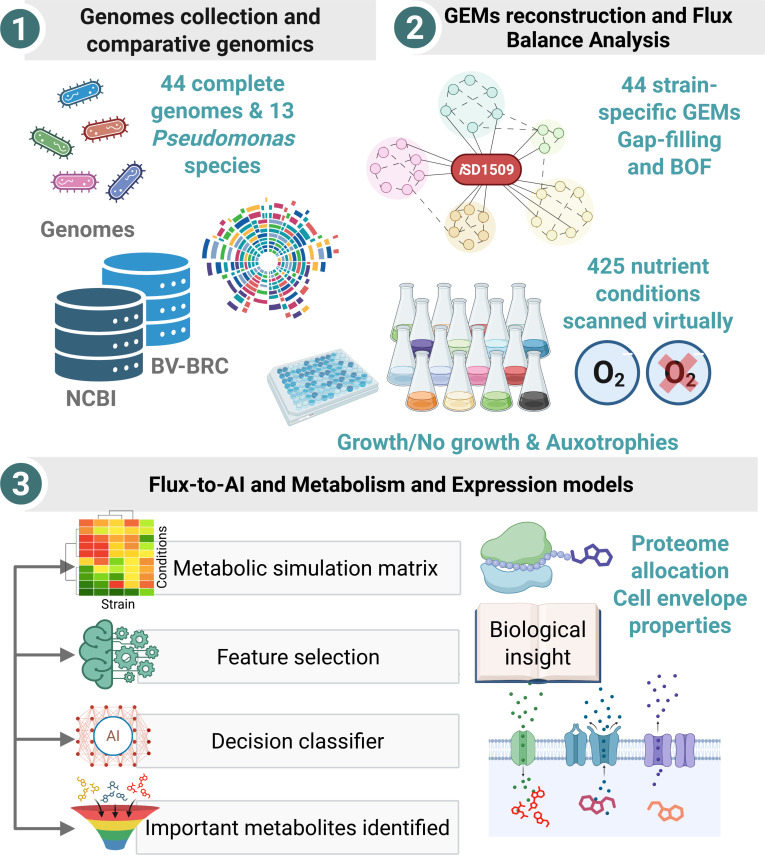

## Introduction

*Pseudomonas* are gram-negative and play vital roles in air, soil, and water ecosystems, as well as in health and disease, due to their metabolic versatility and ecological functions [1]. They are among the most abundant organisms on Earth, according to the Earth Microbiome Project [2]. In soil, they can act as plant-growth-promoting rhizobacteria, enhancing nutrient exchange and availability as well as suppressing pathogens through mechanisms like siderophore production and phytohormone synthesis [3]. In aquatic environments, *Pseudomonas* spp. can contribute to bioremediation by degrading pollutants such as nitrogenated compounds and hydrocarbons and by accumulating heavy metals, thereby improving water quality [4,5]. A crucial example of this is the denitrification process during wastewater treatment, where they convert nitrite (NO_2_^−^) and nitrate (NO_3_^−^) into nitrogen gas (N_2_), a vital step for nitrogen removal and balance in the nitrogen cycle [6]. Their ability to form biofilms and produce biosurfactants aids in the breakdown of hydrophobic compounds, facilitating pollutant dispersion and degradation [7]. Moreover, *Pseudomonas* spp. participate in air treatment by degrading airborne pollutants, thereby contributing to atmospheric detoxification in controlled systems [8]. *Pseudomonas* in clinical settings can be pathogenic, making them particularly interesting, as they have been shown to adapt their metabolism in response to the host [9].

Comparative genomics studies of *Pseudomonas* have been carried out for soil and clinical studies. They have revealed ecological adaptation and functional diversity. For example, genomic determinants of multitrophic interactions in *Pseudomonas fluorescens* and *Pseudomonas* spp. have been identified as key biological control mechanisms that can collectively contribute to the modulation of plant immunity [10,11]. Recently, a large-scale analysis of >3,000 genomes, uncovering host- and niche-specific genetic adaptations across the genus, highlighted substantial differences depending on the origin of *Pseudomonas* [12]. Comparative genomic analysis of *Pseudomonas corrugata* and *P. mediterranea* revealed that virulence in these plant pathogens does not rely on the canonical type III secretion system, but instead is associated with conserved type VI secretion systems, quorum sensing networks, siderophore production, and secondary metabolite biosynthetic gene clusters, demonstrating that pathogenicity can arise through alternative mechanisms adapted to specific ecological niches [13]. Most recently, multiple genomes of clinically relevant *Pseudomonas aeruginosa* were analyzed, identifying carbapenem- and aztreonam-resistance determinants in healthcare settings [14]. Collectively, these studies demonstrate how genomic analysis can decode the strain-specific capabilities of *Pseudomonas* across environments, from soil systems to clinical infections, while highlighting shared and unique genetic features underlying their ecological success.

A major limitation in comparative microbial genomics is that multi-omics datasets (e.g., metabolomics, phenotypic, proteomic, and transcriptomic) are often unavailable for the large number of genomes currently deposited in public repositories. The primary objective of the Flux-to-AI framework is to bridge the genotype-to-phenotype gap by transforming genomic information into mechanistically meaningful metabolic phenotypes using genome-scale constraint-based models (genome-scale metabolic models [GEMs]). Unlike genome-based approaches that rely solely on gene or reaction presence/absence and therefore represent only the potential metabolic repertoire of an organism, flux simulations integrate gene–protein–reaction associations, network topology, stoichiometric constraints, and environmental conditions to predict functional metabolic capabilities. As a result, the generated flux distributions and growth phenotypes serve as scalable proxies for cellular functional states, capturing pathway-level activity and emergent metabolic behaviors that cannot be inferred from individual genes alone. This systems-level representation not only provides biologically interpretable features for machine-learning analyses but also establishes a direct connection to metabolism and expression (ME) models, enabling the investigation of proteome allocation and cell envelope remodeling across strains.

The use of combined systems biology tools for the analysis of biological data is a well-established framework that integrates up-to-date knowledge from the literature and multi-omics data to characterize, understand, predict, and ultimately modulate a wide variety of biological processes in which *Pseudomonas* is involved [15]. One of the main tools for elucidating the characteristics of biological systems is the reconstruction of GEMs [16]. These models are knowledge bases built from genetically, metabolically, and biochemically curated information, allowing researchers to predict the impact of environmental perturbations on the phenotypes of an organism [17]. One of the emerging applications of GEMs is the reconstruction of metabolic models for multiple strains as well as metabolism and translation models, which focus on comparing the diversity in the metabolic characteristics of a species [18–20] and the simulation of protein resource allocation [14,18,21,22]. This strategy is highly useful for classifying strains by the phenotypes they exhibit and the environmental niche in which they develop while accounting for the nutrients and energy required to produce the proteins that carry out metabolic functions.

Multistrain genome-scale metabolic reconstruction is built using highly curated models as templates. For example, the early foundational models of *Pseudomonas*, such as *i*MO1056 for *P. aeruginosa* PAO1 [23] and *i*JP815 for *P. putida* KT2440 [24], successfully mapped core metabolic networks based on primary genome annotations. Subsequent specialized iterations, including *i*Pae1146 [25] and the recent *i*JD1249 [26], introduced highly specific single-strain refinements targeting pathogenic traits, such as quorum sensing pathways and precise amino acid catabolism under localized physiological conditions. These model properties are mostly captured in the latest *P. aeruginosa* model *i*SD1509 [27], which was used as a template for our analysis. These models were developed and successfully applied for single strains; however, interoperability remains challenging. In contrast, multistrain models transcend the single-strain paradigm by capturing phenotypic variations across the *Pseudomonas* pan-genome, translating genetic diversity into a standardized metabolic matrix for high-throughput comparative simulations.

Since the discovery of *Pseudomonas* in 1882, about 2,534 complete genomes have been collected and deposited in the National Center for Biotechnology Information (NCBI) (https://www.ncbi.nlm.nih.gov/) [28] and Bacterial and Viral Bioinformatics Resource Center (BV-BRC) (https://www.bv-brc.org) [29]. Here, we reconstructed GEMs from the most representative and highest-quality *Pseudomonas* genomes. These models suggest strain-specific catabolic capabilities and toxic compound removal under aerobic and anaerobic conditions, highlighting the metabolic diversity of *Pseudomonas* species and how differences in genomic content provide distinct strategies for controlling biomass growth and for performing modeling analyses with models that share identical metadata. By integrating genomic information with biochemical knowledge, GEMs can reveal knowledge gaps in strain-specific metabolism, including incomplete metabolic pathways, unresolved gene-to-reaction associations, and missing functional annotations. These limitations highlight the need for high-quality GEMs to accurately investigate environmentally relevant *Pseudomonas* strains at the systems level.

## Results and Discussion

### Expansion of the *P. aeruginosa* model (*i*SD1509)

Our first goal was to obtain a high-quality *Pseudomonas* GEM to use as a reference for the multistrain reconstruction. We used the recently published *P. aeruginosa* PA14 GEM (*i*SD1509), since it is built on top of previous reconstructions, contains the largest gene content for any *P. aeruginosa* GEM (1,509 genes), and is the most accurate model with 92.4% (gene essentiality) and 93.5% (substrate utilization) prediction accuracies [27]. This model was built mainly to investigate virulence and drug potentiation. Therefore, we tested the model’s ability to degrade xenobiotic compounds (xylene, toluene, and benzene). Since the model was unable to metabolize these compounds, we used the model of *P. putida* KT2440 (*i*JN1463) to add xenobiotic reactions, the model of *Escherichia coli* K-12 substr. *MG1655* (*i*ML1515) to add intermediary reactions [30], and *Nitrosomonas europaea* (*i*GC535) to add nitrification reactions [31], as these models are highly annotated and well curated. We performed a homology search against the template model genomes and extracted the bidirectional best-hit pairs for genes involved in xenobiotic metabolism. Overall, 3 transport reactions and 2 metabolic reactions were added (see Dataset S1). Once the reactions were added, the model was able to grow using xylene, toluene, and benzene as carbon sources under aerobic (M9) conditions. After curation, the model contained 1,514 genes and 2,030 reactions.

### Comparative genomics of *Pseudomonas* strains

**Fig. 1.**
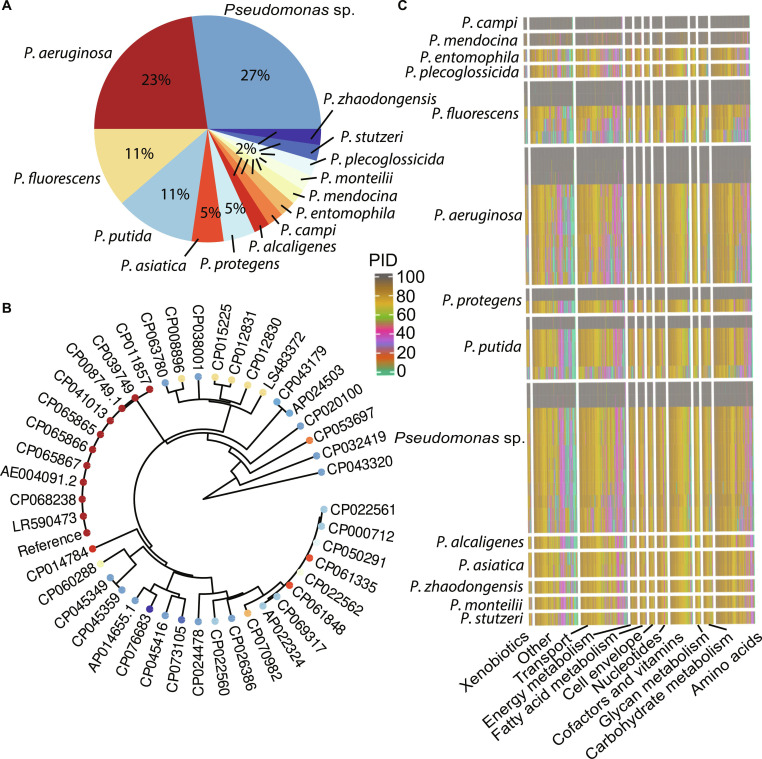
(A) Species distribution of the 44 *Pseudomonas* genomic sequences downloaded from the Pathosystems Resource Integration Center (PATRIC) and National Center for Biotechnology Information (NCBI) GenBank databases. (B) Phylogenetic tree of 44 *Pseudomonas* strains reconstructed from 16S ribosomal RNA (rRNA) sequences. Coloring from panel (A) corresponds to the strains in panel (B). (C) Homology comparison of the *Pseudomonas aeruginosa* PA14 genome *vs* the 44 *Pseudomonas* strain genomes using Basic Local Alignment Search Tool (BLAST). Rows are clustered by subsystem for the reference model genes. In columns are *Pseudomonas* genomes grouped by species. The color code indicates the percentage of identity (PID).

To generate a representative dataset for comparative metabolic analysis, 44 *Pseudomonas* genomes were selected from the BV-BRC and NCBI GenBank databases based on genome completeness, annotation quality, and representation of the phylogenetic and ecological diversity of the genus. Priority was given to complete or high-quality draft genomes with publicly available annotations and broad representation of species commonly associated with clinical, soil, aquatic, and wastewater environments. When multiple genomes from the same species were available, strains were selected to capture intraspecies diversity while minimizing redundancy. This collection provides a standardized dataset suitable for reconstructing strain-specific GEMs and systematically comparing their predicted metabolic capabilities across diverse environmental niches. The breakdown across 13 species is shown in Fig. 1A and B, with *Pseudomonas* sp., *P. aeruginosa*, *P. putida*, and *P. fluorescens* having the highest numbers of genomes in the dataset.

The phylogenetic tree showed that the *P. aeruginosa* strains were grouped with the reference strain PA14, as expected, since they belong to the same species (Fig. 1B). Near the *P. aeruginosa* cluster, we found several *Pseudomonas* sp. strains grouped with *P. fluorescens*, suggesting a closer phylogenetic relationship. We also observed that other *Pseudomonas* sp. strains clustered with *P. zhaodongensis* and *P. stutzeri*, highlighting the phylogenetic diversity among the unclassified isolates. This clustering may reflect taxonomic uncertainty or incomplete species assignment rather than distinct evolutionary trajectories, emphasizing the genetic heterogeneity present within the *Pseudomonas* sp. group. Finally, *P. putida* strains formed the most distant group, together with *P. protegens*, *P. asiatica*, *P. plecoglossicida*, and *P. monteilii*.

The 44 genomic sequences were bidirectionally compared against the *Pseudomonas* reference genome using Basic Local Alignment Search Tool (BLAST). The percentage of identity (PID) of the reference model genes was extracted from the results of these comparisons, resulting in a homology matrix of 1,514 (model genes) × 44 (*Pseudomonas* genomes). The model genes were annotated according to the subsystem in which they participate, and a heat map was generated with the PID (Fig. 1C). The *P. aeruginosa* genomes exhibit the highest rates of identity (PID) relative to the reference genome, with some genomes showing variation in their PID, particularly in genes associated with transport functions. This is expected since the genomes belong to strains of the same species. Previous pan-genomic studies have estimated a size of between 655 and 2,500 genes for the *P. aeruginosa* core genome [32,33], so it is expected that the number of orthologous genes between strains falls within this range. Likewise, it has been shown that the size of the core genome varies with the number of strains included in pan-genome reconstructions [33,34], such that as the sample size increases, the core genome decreases, and vice versa.

Regarding the other *Pseudomonas* species, fewer orthologous genes were observed in the transport category, followed by genes related to amino acid and carbohydrate metabolism. This difference in genomic content is consistent with what has been reported for *P. putida* strains [35], which may be due to the different metabolic capacities associated with the lifestyles and environments in which the species develop [36]. Based on the comparative homology analysis (Fig. 1C), genes associated with cofactor and vitamin metabolism, nucleotide metabolism, and terpenoid biosynthesis exhibited consistently higher sequence conservation than many other functional categories across the analyzed strains.

### Reconstruction of strain-specific GEMs

To systematically compare the metabolic potential of diverse *Pseudomonas* species, strain-specific GEMs were reconstructed from a curated reference model using homology-based gene transfer followed by model refinement and quality assessment. First, the effect of ortholog stringency on GEM reconstruction was assessed using 3 amino acid sequence identity (PID) thresholds: 60%, 70%, and 80%. These thresholds represent moderate to stringent homology criteria and span the range commonly explored in homology-based bacterial GEM reconstruction; evaluating multiple identity thresholds provides a systematic assessment of the trade-off between orthology confidence and model completeness. At a PID value of 80%, only 9 models (corresponding to *P. aeruginosa*) had more than 90% of the genes from the reference model, while the rest of the models had less than ~45%. At a PID value of 70%, 38 models had more than 50% of the genes. Finally, at a PID value of 60%, 41 preliminary models had more than 50% of the genes. Based on this analysis (Fig. S1), the 60% PID threshold was selected for the final reconstructions because it provided the best balance among preserving orthologous gene content, maintaining model functionality, and minimizing the need for extensive gap filling across the diverse *Pseudomonas* species analyzed.

All present genes (from the reference genome) were mapped to the gene locus of the ortholog gene (from strains), and the gene–protein–reaction rule was modified accordingly. A gene essentiality analysis was performed in each strain model for all the external genes (M9 and Luria–Bertani [LB] medium). The nonessential reactions associated with those genes were eliminated from the models (Dataset S1). After minimizing the number of external genes and performing functional evaluation, all strain-specific models were able to grow on M9 and LB media. We used MEMOTE [37] to validate our models, and all models scored above 81.9%, with a median of 85.1% (see Materials and Methods for more details).

The biomass objective function (BOF) of each reconstructed model was imported from the well-curated *i*SD1509 model. This approach follows current best practices in comparative genome-scale metabolic modeling by providing a standardized biomass formulation across all strain-specific models, thereby enabling direct comparison of predicted metabolic phenotypes while minimizing methodological variability. However, experimentally determined differences in biomass composition, particularly in cell envelope components, membrane lipids, and macromolecular composition, may influence quantitative flux distributions in phylogenetically distant species.

### GEM-predicted growth capabilities differentiate across strains

**Fig. 2.**
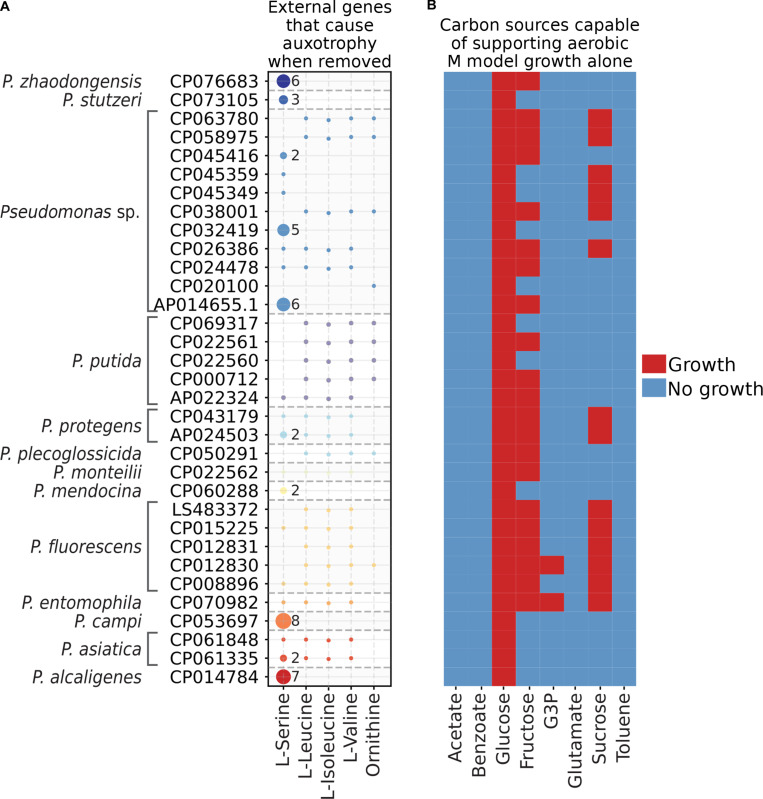
(A) Potential auxotrophies for the 44 strain-specific genome-scale metabolic models (GEMs). Rows are the GenBank accessions for the strains. Columns represent the nutrient for which the strains are auxotrophic. The *Pseudomonas aeruginosa* model had no additional auxotrophies; thus, it is not plotted. (B) Predicted growth capabilities of the 44 strain-specific *Pseudomonas* GEMs. The simulations were performed in M9 minimal medium (Data S1) under aerobic conditions. Uptake rates were constrained to 17 and 20 mmol g DW^−1^ h^−1^ for the carbon sources and oxygen, respectively. The *Pseudomonas* species and their GenBank accession numbers are in rows. Carbon source tested in columns. Color coding: red = growth; blue = no growth.

**Fig. 3.**
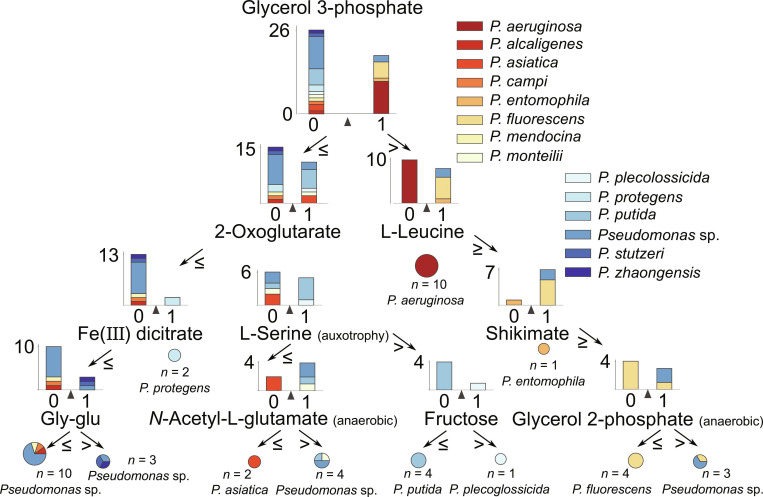
Decision tree classifier of *Pseudomonas* species based on the 405 simulated conditions across 44 strain-specific models. Bar plots represent the classes that are split by the decision tree based on the ability of the models to grow on each substrate. Arrows show the outcome of the decision. Pie charts at the bottom of the decision tree represent the final classification of the models, showing the most representative class as the name of the pie chart. Bar plots show all metabolites broken down on the *x*-axis of several trees, and on the *y*-axis the number of times that they appeared, and are sorted by appearances.

Once we obtained the functional strain-specific models, we evaluated their ability to reproduce known *Pseudomonas* phenotypes (Figs. 2 and 3). We simulated aerobic and anaerobic growth using acetate, glucose, fructose, sucrose, and glutamate as carbon sources for validation [38–41]. We also simulated the degradation of xenobiotic compounds (toluene and benzene) as these phenotypes are reported in a wide range of *Pseudomonas* species [42–44]. Under aerobic conditions, *P. fluorescens* and *P. alcaligenes* showed the lowest versatility in substrate utilization, growing only with glucose, fructose, glycerol, and sucrose, suggesting that these strains are less adaptable to environmental nutritional changes. On the other hand, *P. aeruginosa*, *P. putida*, *P. protegens*, and *P. zhaodongensis* exhibited the greatest versatility, growing on most of the carbon sources tested and degrading toluene and benzene.

These results show strain-specific capabilities, with strains within the same species exhibiting variation in their ability to utilize alternative carbon sources. High metabolic versatility has been described previously in *Pseudomonas* [35], and the utilization of alternative carbon sources has been directly associated with the capacity to survive in different nutritional environments, allowing them to adapt to starvation conditions and to host–pathogen environments [45]. In the environment, *Pseudomonas* strains can even thrive, utilizing nitrogen and other toxic compound emissions [46–48] from the agricultural industry and the discharge of industrial and domestic wastewater as one of the primary sources of pathogens for the infection chain [48,49]. We also observed that high metabolic versatility in *Pseudomonas* is related to the ability to degrade xenobiotic compounds [19], which could be an indication of the lifestyle of the strains [36]. Under anaerobic conditions, most of the strains were able to grow with glucose, fructose, glycerol, and sucrose. Interestingly, growth on glutamate was observed in a low number of strains (mainly from *P. aeruginosa*), and no growth was predicted in any of the strains on toluene, benzene, or acetate.

The auxotrophies and carbon source utilization profiles identified in this workflow represent model-predicted traits rather than experimentally validated phenotypes; as such, these *in silico* capabilities may reflect gaps in vertical genome annotation, PID threshold constraints, or missing metabolic pathways in the drafts. These simulated auxotrophic requirements closely mirror the nutrient-depleted regimes observed in specific clinical niches, providing a computational parallel to metabolic pressures found in cystic fibrosis (CF) sputum. However, the biological complexity of microorganisms and the lack of understanding of the metabolic capabilities involved in the processing of nitrogen compounds represent a limitation to optimizing these treatments [50]. Importantly, this pipeline is a strictly computational analysis and can generate testable hypotheses about strain-specific evolutionary niches.

### GEMs enable the investigation of species-specific auxotrophies

**Fig. 4.**
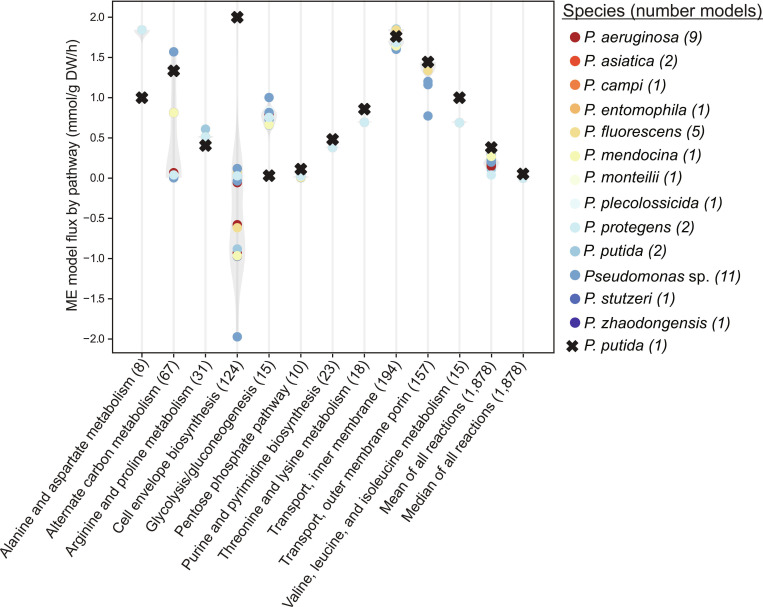
Predicted fluxes using metabolism and expression (ME) models. Difference in standardized flux per subsystem between the metabolic (M) and ME models for each strain. The number of reactions in each subsystem is given. Results are colored by the species of each model. Only the subsystems that change the most are shown. The manually curated ME model of *Pseudomonas putida* is included for comparison (labeled *P. putida*; available at https://github.com/jdtibochab/pputidame/tree/master/model). Constraints were the same across all models, and only strain-specific genome sequences varied for each model.

To better understand the nutritional preferences of the species, we used the strain-specific GEMs to examine the presence and the genetic basis of potential auxotrophies (see Materials and Methods). By removing external genes one by one in the strain-specific models, we observed that several models failed to produce at least one biomass constituent on M9 minimal medium supplemented with glucose. Therefore, we investigated the impact of adding extracellular nutrients to the models under these conditions. Of the 20 nutrients added, we found potential auxotrophies for only 5: l-isoleucine, l-leucine, l-serine, l-valine, and ornithine (Fig. 4). Rather than a species-wide trend, l-serine auxotrophy was observed in specific, isolated strains of *P. zhaodongensis*, *P. campi*, and *P. asiatica* and 7 of the 11 *Pseudomonas* sp. strains, while *P. alcaligenes* retained alternative pathways. Similarly, the absence of the *ilvE* gene in *P. putida* R51 is hypothesized to reflect a metabolic adaptation to specialized environments such as wastewater, although experimental evidence is required to confirm this evolutionary pressure. We further investigated the gene responsible for this auxotrophy and identified PA14_66260 (*ilvE*, encoding a branched-chain amino acid [BCAA] aminotransferase) as the cause. This gene is associated with leucine, valine, and isoleucine transaminase reactions (LEUTAi, VALTA, and ILETA). We did not find an *ilvE* ortholog for this strain. The only candidate showed a ~30% PID; thus, it was kept as an external gene in the model.

Amino acid auxotrophs have been reported previously in *Pseudomonas*, and it has been shown that these nutrients are provided from the environment, especially in the high amino acid content of sputum from the CF lung environment [51,52]. In gram-positive bacteria, BCAA auxotrophies have been primarily associated with host–pathogen interactions [53] and coordination of virulence expression [54]. On the other hand, leucine auxotrophy has been shown to provide a fitness advantage in *P. fluorescens* in soil environments [55]. In *P. putida*, *ilvE* acts as the primary transaminase in BCAA degradation and biosynthesis, making it essential for growth on minimal medium [56,57], consistent with the phenotype predicted by our *P. putida* R51 GEM. Interestingly, this strain was isolated from wastewater and exhibits both antibiotic resistance/degradation and heavy-metal resistance [58]. These traits, together with the GEM-predicted auxotrophy, indicate the metabolic adaptation of the strain to a nutrient-rich but heavily contaminated environment, such as wastewater.

However, there is a lack of studies on auxotrophic strains from wastewater environments, with only a recent study identifying auxotrophic *Pseudomonas* strains isolated from a sulfur-based denitrification system [59]. Therefore, the potential auxotrophies identified here could serve as a starting point for studying the nutritional requirements and preferences of *Pseudomonas* strains in wastewater. Since microbial communities are often present in wastewater treatment systems [60], such studies could facilitate our understanding of potential interactions between *Pseudomonas* and other community members.

It is important to consider that GEMs predict the metabolic potential of an organism under defined environmental conditions by identifying substrates that support growth through the reconstructed metabolic network. Accordingly, the ability of a model to utilize aromatic or toxic compounds should be interpreted as a predicted metabolic capability rather than a direct measure of biodegradation rate, pollutant removal efficiency, or toxicity tolerance. The latter depend on additional physiological and environmental factors, including gene regulation, enzyme expression, transport kinetics, cosubstrate availability, and oxygen conditions, which are beyond the scope of the current constraint-based modeling framework. Nevertheless, these predictions provide mechanistic hypotheses for identifying candidate metabolic pathways and strains for subsequent experimental evaluation.

### Applying the Flux-to-AI approach to identify transport and catabolic capabilities that differentiate *Pseudomonas* species

Since we previously detected differences in carbon source utilization and auxotrophies across species and strains, we hypothesize that these abilities may reflect functional differences in their capacity to survive in different nutritional environments. Therefore, we aimed to develop a classification schema for *Pseudomonas* species based on their GEM-predicted metabolic capabilities. To achieve this, we simulated biomass production across over 405 nutrient conditions by testing growth on all carbon sources available in the models under both aerobic and anaerobic conditions. These results were used as features for species classification (Fig. 3).

By building the classification schema of *Pseudomonas*, we found that the ability to grow on glycerol 3-phosphate (G3P) separated almost half of the strains analyzed, with 18 and 26 strains growing and not growing with G3P, respectively. Growth on G3P and l-leucine as sole carbon sources differentiate *P. aeruginosa* species from the rest. Interestingly, studies have shown that G3P homeostasis affects growth and virulence factor production in *P. aeruginosa* PAO1 and that its metabolism contributes to adaptation and persistence in the CF lung environment [61,62]. Similarly, it has been shown that l-leucine catabolism may be associated with the establishment of infections by *P. aeruginosa* strains in CF patients [63]. The presence of pathogenic and antibiotic-resistant strains in wastewater effluents has been a concern in recent years [64], since the microenvironment in wastewater treatment plants can act as a reservoir of antibiotic resistance [65].

Given the challenges of balancing features, we identified the minimal set of metabolic features with the highest predictive power by implementing a biology-driven feature selection strategy based on iterative network perturbation rather than conventional dimensionality-reduction approaches such as principal component analysis or partial least squares. These methods can reduce the biological interpretability of flux-derived features by projecting them into latent variables, and partial least squares further assumes linear relationships. In contrast, our classification was performed on binary growth phenotypes that do not necessarily satisfy this assumption. Individual reactions were systematically removed from the 405 simulated metabolic features, and the resulting decision tree topology was re-evaluated after each perturbation to assess the contribution of every feature to species classification. This iterative elimination process identified a subset of peripheral exchange reactions under aerobic and anaerobic conditions, including EX_3mb_e, EX_3oxoadp_e, EX_acon_C_e, EX_gsn_e-ana, and EX_gua_e-ana, whose removal produced no changes in the decision tree architecture, indicating that these reactions were redundant and could be excluded without compromising classification performance (see Dataset S1). Overall, cross-validation was performed using *K*-fold with 5 splits, resulting in the following: accuracy, avg 0.8394 and SD 0.03533; F1 macro, avg 0.82657 and SD 0.05560; and recall macro, avg 0.83998 and SD 0.03266. To evaluate whether the decision tree was biased by unequal species representation, we repeated the analysis using a balanced subset of genomes from the 5 most represented *Pseudomonas* groups (Supplementary Materials). The resulting classifier identified many of the same central metabolic capabilities, including aromatic compound metabolism, ribose, aconitate, isocitrate, and iron acquisition, indicating that the principal discriminative metabolic features are robust to changes in species composition.

The GEM-predicted metabolic capabilities of *Pseudomonas* could serve as a starting point for the further examination of pathogenic and antibiotic-resistant strains in wastewater. Furthermore, *P. fluorescens* and some strains of *Pseudomonas* sp. are distinguished by their inability to utilize leucine and shikimate for growth. At the same time, *P. entomophila* can grow with shikimate as a main carbon source. We also identified that the presence of an l-serine auxotrophy differentiates *Pseudomonas* sp. and *P. asiatica* from *P. putida* and *P. plecoglossicida*. These results highlight the diversity of nutritional environments in which different *Pseudomonas* species can thrive [35,36,52], as most species are clearly differentiated by their presence or absence of metabolic capabilities. *Pseudomonas* sp. exhibited the highest metabolic diversity, indicating that these strains can adapt to a wide range of nutrient niches. To further understand the AI-unraveled metabolic and transport capabilities, we built resource allocation models to assess whether changes in genome sequence could explain variation in predicted phenotypes across *Pseudomonas* strains.

Although phylogenetic relatedness is expected to influence the metabolic repertoire of closely related *Pseudomonas* strains, the Flux-to-AI framework was designed to classify strains based on predicted metabolic phenotypes rather than directly on genomic similarity. The machine-learning model identified a limited set of nutrient utilization capabilities that remained robust after iterative feature elimination and whose biological relevance was independently supported by ME model simulations. These findings suggest that the classifier captures functionally relevant metabolic adaptations associated with environmental niche specialization in addition to the evolutionary relationships among strains. Nevertheless, we recognize that completely disentangling phylogenetic effects from functional convergence will require larger, phylogenetically balanced datasets and experimental phenotypic validation across diverse *Pseudomonas* lineages.

### Strain-specific genome sequences that drive changes in cell envelope were found using ME models

To further investigate the biological basis of the metabolic capabilities identified by the Flux-to-AI framework, we analyzed the corresponding pathways using ME models. ME models incorporate the transcription and expression machinery into metabolic (M) models, allowing for more accurate predictive capabilities tailored to strain-specific genome sequences and proteome allocation [21]. coralME can be used to semiautomatically generate ME models from M models, enabling us to expand our analysis to predict gene expression requirements based on strain-specific sequences (Table 1) [66]. We generated ME models for each *Pseudomonas* M model and performed simulations in M9 minimal medium supplemented with glucose. The predicted solutions for the M and ME models differed greatly, as evidenced by large changes in reaction fluxes (Fig. 4). All metabolites and reactions can be identified using the Supplementary Materials and in the BiGG Database [67]. Flux model predictions were standardized using previously published normalization strategies [68], in which pathway fluxes are divided by the predicted growth rate. ME models have historically been built using large amounts of custom annotations and literature research; therefore, for comparison, the manually curated *P. putida* model (black cross) was included in the analysis [69]. Results showed that flux distributions from our *Pseudomonas* models are comparable to those from manually curated ME models.

**Table 1.** Number of reactions, metabolites, and genes in the M and coralME-generated ME models

Strain	M model reactions	M model metabolites	M model genes	ME model reactions	ME model metabolites	ME model genes
CP008896	1,764	1,648	1,069	9,533	5,484	1,012
CP053697	1,591	1,648	844	8,064	4,794	821
CP026386	1,743	1,648	1,055	9,436	5,456	1,006
CP069317	1,688	1,648	969	8,655	5,082	918
AE004091.2	2,017	1,648	1,467	12,362	6,757	1,435
CP014784	1,634	1,648	876	8,346	4,927	849
CP073105	1,629	1,648	874	8,148	4,886	833
CP065866	2,024	1,648	1,489	12,529	6,840	1,457
CP061848	1,739	1,648	1,057	9,438	5,441	1,006
CP012831	1,756	1,648	1,083	9,470	5,461	1,020
CP008749.1	1,997	1,648	1,468	12,377	6,774	1,419
CP032419	1,690	1,648	957	8,855	5,136	921
CP038001	1,788	1,648	1,110	9,699	5,559	1,048
CP022560	1,743	1,648	1,056	9,379	5,421	996
CP076683	1,628	1,648	855	8,024	4,802	809
CP045416	1,599	1,648	848	7,976	4,781	799
AP022324	1,750	1,648	1,060	9,444	5,429	1,001
LS483372	1,764	1,648	1,083	9,580	5,494	1,027
CP022562	1,732	1,648	1,049	9,338	5,403	989
LR590473	2,011	1,648	1,474	12,465	6,794	1,430
CP065865	2,019	1,648	1,487	12,550	6,835	1,455
CP058975	1,768	1,648	1,092	9,386	5,421	1,022
CP012830	1,772	1,648	1,080	9,572	5,518	1,031
AP014655.1	1,597	1,648	845	7,905	4,760	792
CP061335	1,735	1,648	1,048	9,288	5,384	993
CP068238	1,997	1,648	1,412	11,985	6,604	1,379
CP050291	1,724	1,648	1,019	9,208	5,354	975
CP070982	1,738	1,648	1,049	9,364	5,424	994
CP000712	1,738	1,648	1,046	9,366	5,394	986
AP024503	1,801	1,648	1,130	9,943	5,660	1,080
CP045349	1,688	1,648	981	8,976	5,195	934
CP063780	1,774	1,648	1,072	9,532	5,485	1,025
CP024478	1,719	1,648	989	9,017	5,264	868
CP022561	1,745	1,648	1,047	9,398	5,405	990
CP065867	2,019	1,648	1,479	12,496	6,817	1,447
CP020100	1,455	1,648	654	6,418	4,036	599
CP039749	2,024	1,648	1,487	12,585	6,877	1,455
CP041013	2,024	1,648	1,488	12,533	6,837	1,456
CP015225	1,746	1,648	1,071	9,374	5,413	1,001
CP045359	1,688	1,648	981	8,971	5,191	934
CP043320	1,379	1,648	591	5,957	3,879	549
CP060288	1,683	1,648	979	8,884	5,159	933
CP043179	1,828	1,648	1,157	10,133	5,746	1,109

M model, metabolic model; ME model, metabolism and expression model

The ME model simulations showed consistent changes in protein allocation associated with the metabolic features identified by the decision tree, particularly within amino acid, cofactors, and cell-envelope-associated pathways. These results provide mechanistic support for Flux-to-AI predictions by demonstrating that the metabolic capabilities distinguishing *Pseudomonas* strains are accompanied by coordinated changes in cellular resource allocation, thereby strengthening the biological interpretation of the computationally predicted phenotypes.

To quantify the pathway-level differences predicted by the ME models, we compared the average change in flux between the strain-specific ME models and the manually curated *P. putida* reference model (Fig. 4 and Supplementary Materials). The largest positive average increases were observed in alanine and aspartate metabolism (1.82 mmol g DW^−1^ h^−1^), inner membrane transport (1.75 mmol g DW^−1^ h^−1^), and cysteine metabolism (1.46 mmol g DW^−1^ h^−1^), indicating substantial differences in amino acid metabolism and transport resource allocation among strains. In contrast, pathways associated with glycerophospholipid metabolism (−0.83 mmol g DW^−1^ h^−1^), cell envelope biosynthesis (−0.55 mmol g DW^−1^ h^−1^), carbohydrate metabolism (−0.49 mmol g DW^−1^ h^−1^), and cofactor and vitamin metabolism (−0.43 mmol g DW^−1^ h^−1^) exhibited negative fluxes in remarkable amounts, which are usually associated with reversible reactions. Most remaining pathways showed relatively small average changes (<0.2 mmol g DW^−1^ h^−1^), indicating that the major proteome allocation differences are concentrated within a limited number of metabolic subsystems rather than being uniformly distributed across the metabolic network. Although the median flux change across all reactions remained close to zero, several pathways displayed broad variability among species, particularly those involved in membrane transport and cell-envelope-associated metabolism, supporting the interpretation that strain-specific metabolic capabilities are accompanied by distinct resource allocation strategies.

Overall, the ME models show higher flux through amino acid-related pathways compared to the M models, likely due to the amino acid demand created by the additional ME model requirements for enzyme and messenger RNA costs. Interestingly, the ME models show comparatively lower activity in cell envelope pathways despite higher activity in transport genes. This variability appears to be independent of the number of reactions in the subsystem, as some pathway fluxes remain unchanged regardless of subsystem size. The literature supports this model-generated hypothesis, as *Pseudomonas* has been shown to contain very few general porins and instead uses highly specific import porins, likely a factor in its high drug and stress tolerance [70]. These changes can confer different metabolic capabilities and differentiate between environments and pathogenicity [71,72].

Here, we develop the most comprehensive resource for *Pseudomonas* available to date. Genome-scale and ME models were reconstructed for 44 strains in accordance with high-quality standards. Models are now available to perform comparative analysis, overcoming model compatibility while ensuring high prediction accuracy. Model simulations enabled the unraveling of how cell envelope composition changes resource allocation in *Pseudomonas* and how the metabolism of G3P and some amino acids drives how they thrive under various scenarios.

## Conclusion

*Pseudomonas* species exhibit remarkable metabolic versatility that underlies their ability to thrive across diverse environmental, clinical, and industrial niches. In this study, we developed a Flux-to-AI framework that integrates comparative genomics, genome-scale metabolic modeling, machine learning, and ME modeling to investigate how genomic diversity translates into strain-specific metabolic phenotypes. By reconstructing GEMs for 44 *Pseudomonas* strains and simulating growth under 425 nutrient conditions, we identified key metabolic capabilities that differentiate species and reveal distinct strategies for substrate utilization. Complementary ME model analyses provided mechanistic support by demonstrating that these metabolic differences are accompanied by changes in proteome allocation across central metabolic, transport, and cell-envelope-associated pathways. Together, the models, computational workflow, and analyses presented here establish a standardized resource for investigating functional diversity within the genus *Pseudomonas* and provide a foundation for future studies of microbial communities, environmental adaptation, and biotechnological applications.

## Materials and Methods

The Flux-to-AI framework integrates genome-scale metabolic modeling and machine learning to systematically investigate strain-specific metabolic capabilities across the *Pseudomonas* genus. Previously validated steps for multistrain metabolic modeling were followed [36,73–75]. First, genomic information was selected, and then a manually curated reference GEM was expanded and used as a template to reconstruct strain-specific GEMs through comparative genomics. The reconstructed models were subsequently validated and simulated under standardized environmental conditions to predict metabolic phenotypes across a large set of nutrient utilization scenarios. These steps reduce feature dependence and collinearity in downstream machine-learning analyses. These predicted phenotypes were then used as biologically interpretable features for machine-learning classification, followed by feature robustness analyses and ME model simulations to investigate the mechanistic basis of the metabolic capabilities identified by the classifier.

### *Pseudomonas* genome sequence selection

The *Pseudomonas* genomes used for the reconstructions were downloaded from the BV-BRC database [29] and NCBI GenBank [28]. The search was performed for *Pseudomonas* species, and genomes with “complete” status were selected. The results were filtered based on genome metadata using keywords related to nitrogen removal and wastewater treatment processes. Some of the keywords used were “wastewater”, “denitrification”, “nitrate”, “activated sludge”, “ammonium”, and “ammonia”. All data downloaded were cleaned and checked for duplicate entries, resulting in 44 unique genomic sequences. To ensure consistency in gene prediction and annotation, all genomic sequences were reannotated using Prokka 1.14.6 [76].

### Multistrain GEMs reconstruction and expansion of *i*SD1509

The latest model *i*SD1509 of *P. aeruginosa* PA14 was used as a reference model for the strain-specific reconstructions [27]. *i*SD1509 was built based on *i*Pau21 [77]. *P. aeruginosa* models have been improving since 2017, reaching MEMOTE scores of 90% of gene essentiality prediction and 93% of carbon source prediction and containing the highest number of genes (1,509) of any *Pseudomonas* model available to date. Potential limitations associated with a single template can arise, such as the underrepresentation of certain lineage-specific pathways in distantly related species; however, only previously curated GEMs exist for *P. putida*, and only draft models built from *P. aeruginosa* are available for the other 12 strains in this study. This computational study focuses on using a standardized template matrix to evaluate metabolic flux boundaries in simulated environments, serving as a source for microbiome and multiorganism communities of *Pseudomonas* present in wastewater.

The aerobic and anaerobic biomass objective functions from *i*SD1509 were implemented as standardized growth constraints across the genus models. A comparative inspection confirmed that core macromolecular coefficients remain highly standardized, while *i*SD1509 properly accounts for authentic genus-specific innovations, including specialized cyclopropanic lipids (e.g., clpn_pa_17_0_cyc_c) and lipopolysaccharide cores (PA_core_lipidA_c). This baseline ensures that downstream machine-learning classification reflects authentic flux distributions, a result that can be enhanced using experimentally determined information.

After selecting the template model, we performed several initial simulations on *i*SD1509, and the model was unable to grow on sucrose and xenobiotic compounds (benzene, toluene, and xylene) or to perform denitrification. Thus, some model reactions were added through manual curation to fill these gaps using UniProt (https://www.uniprot.org/), Kyoto Encyclopedia of Genes and Genomes (https://www.genome.jp/kegg/), InterPro, PseudomonasDB, and models of *P. putida* KT2440 (*i*JN1463), *E. coli* K-12 substr. *MG1655* (*i*ML1515), and *N. europaea* (*i*GC535). All reactions and metabolites added were checked for mass and charge balance using the COBRA Toolbox function (checkMassChargeBalance) [78]. Details about the reactions and metabolites are viewable at http://bigg.ucsd.edu/ [67]. Default uptake rates were used for reactions unless otherwise noted to enable more direct comparison between different model solutions. After curation, *i*SD1509 was able to grow on sucrose, degrade the xenobiotic compounds, and perform denitrification under anaerobic conditions. For multisubunit complexes, we mapped the exact reference gene–protein–reaction rules (preserving “AND” logic for complexes and “OR” logic for isoforms) directly to the target strain locus tags. This was followed by a functional verification phase in which nonessential reactions associated with genes that did not contribute to growth phenotypes in simulated M9 (aerobic and anaerobic) and LB (aerobic) media were systematically eliminated.

This iterative curation process was evaluated using MEMOTE [37]. MEMOTE provides a standardized assessment of GEMs by examining annotation completeness, stoichiometric consistency, metabolite and reaction annotations, biomass formulation, and overall network integrity. Scores were generated using the default metrics, but the best gene annotation score was used instead of averaging all gene annotation scores. All reconstructed models achieved MEMOTE scores above 81.9%, with a median score of 85.1%, indicating that the models are well curated and of sufficient quality for comparative constraint-based metabolic simulations.

Following the expansion of the reference model, the genome of *P. aeruginosa* UCBPP-PA14 was downloaded from BV-BRC with RefSeq ID (NC_008463.1) [29]. All 44 strain-specific genomic sequences were BLASTed against the reference genome. From the BLAST results, bidirectional best hits were extracted using a PID threshold of 60%. Multistrain genomes were reconstructed according to a previously published protocol [20]. In brief, the BLAST results were parsed into a homology matrix with *i*SD1509 genes in rows and strain genome IDs in columns. The matrix was binarized into a gene presence/absence matrix using the PID threshold. The information in the homology matrix was used to reconstruct draft strain-specific models using the COBRApy package [78]. Present genes were mapped against *i*SD1509 model genes using the function rename_genes(), and absent genes were kept in the models (referred to as external genes) for further inspection. Once the draft models were reconstructed, all external genes were knocked out individually, followed by growth simulations on M9 medium under aerobic and anaerobic conditions, with glucose as the primary carbon source. For aerobic simulations, the oxygen uptake rate was constrained to 20 mmol g DW^−1^ h^−1^ and nitrate uptake rate to zero, and vice versa for anaerobic simulations. If the draft model produced biomass (biomass objective > 0.001), the external gene was removed from the model along with its associated reactions; otherwise, the external gene was kept and flagged for further inspection. Both the aerobic and anaerobic biomass reactions from *i*SD1509 were used in the strain models.

### Determination of the metabolic capabilities of strains

Multiple medium compositions (M9, LB, morpholinepropanesulfonic acid, and synthetic cystic fibrosis sputum medium) were obtained from previously reported reconstructions [27,35]. The bounds of the corresponding exchange reactions for the metabolites in the medium composition were set to default values (−1,000, 1,000) except for the carbon source (17 mmol g DW^−1^ h^−1^) and oxygen and nitrate uptake rates (20 g DW^−1^ h^−1^). Default uptake rates were applied unless otherwise specified to provide a standardized simulation framework, enabling direct comparison of metabolic phenotypes across reconstructed models while minimizing variability introduced by strain-specific transport parameters.

As previously reported by Dahal *et al.*, in anaerobic simulations, all reactions associated with terminal oxidases (if present in the strain model) were constrained to zero at both lower and upper bounds. When testing growth under aerobic and anaerobic conditions, the model objective was set to the aerobic and anaerobic biomass reactions, respectively [27]. All medium functions used in this study are available in Dataset S1 as a Python script. Seven carbon sources (acetate, benzene, glucose, fructose, glycerol, glutamate, sucrose, and toluene) were tested for growth in all strain-specific models, under aerobic and anaerobic conditions using the 4 medium compositions. If the biomass production was >0.001, the model was considered to grow.

### Detection of potential auxotrophs and decision tree building

To detect potential auxotrophs in the strain-specific models, all external genes were iteratively knocked out using the knock_out() function from COBRApy. For each gene knockout, we tested growth on glucose-supplemented M9 minimal medium under aerobic and anaerobic conditions. If the model could not simulate growth, that external gene was flagged for further inspection. Then, essential amino acid supplementation was simulated by setting the corresponding exchange reaction lower bound to −50 mmol g DW^−1^ h^−1^. This value was chosen as a conservative, nonlimiting uptake constraint that permits amino acid availability without imposing the effectively unrestricted uptake limits (−1,000 mmol g DW^−1^ h^−1^) commonly used in other *Pseudomonas* GEMs [23,25].

If the model presented growth, we stopped adding nutrients and minimized the number of import fluxes using the minimal_medium() function, with the option to minimize components. The models were classified as potential auxotrophs for the resulting amino acids, with the genes identified as the source of the auxotrophy.

Finally, we simulated growth on all carbon sources available in the models by setting their lower bound to −50 mmol g DW^−1^ h^−1^, and constraints for M9 minimal medium under aerobic and anaerobic conditions. We combined the results of these simulations with the predicted auxotrophies, yielding a total of 405 nutritional environments tested using the strain-specific models. These capabilities were used as features for classifying strain-specific models across different *Pseudomonas* species. The classification schema was built using a decision tree classifier from scikit-learn, and the resulting tree was visualized with the dtreeviz library [79]. For cross-validation of the decision tree training, we used the StratifiedKFold class from scikit-learn, with the parameters n_splits = 5, shuffle = True, and random_state = 42, and evaluated testing accuracy, F1 macro, and recall macro.

### ME model creation and simulation

ME models were created using coralME (coralme.readthedocs.io/en/latest/), and the resulting models were placed in a GitHub repository specifically for this article (https://github.com/cristalzucsd/Pseudomonas). coralME version 1.1.5 uses M models and a genome annotation file as input to generate an ME model from this information. Solutions were run, MEMOTE was performed, and results were plotted in this paper using the Jupyter Notebooks available at https://github.com/cristalzucsd/Pseudomonas/tree/main/notebooks. Models were simulated using COBRApy 0.26.3 for Python 3.10. Simulations were performed using the Quad MINOS software [80]. The Quad MINOS solver was compiled under Ubuntu 22.04 with gfortran version 5 and Python 3.10 (pip 22.3.1, wheel 0.38.4, numpy 1.21.6, scipy 1.7.3, cython 0.29.32, and cpython 0.0.6). Fluxes were normalized according to Zuñiga *et al.* [68].

## Data Availability

All genome-scale metabolic models, metabolism and expression (ME) models, Python scripts, Jupyter notebooks, and data generated or analyzed during this study are publicly available in the GitHub repository at https://github.com/CZLaboratory/Pseudomonas. The reconstructed models, simulation scripts, and supplementary datasets required to reproduce the analyses are also provided as Supplementary Materials. Publicly available genome sequences used for model reconstruction were obtained from the BV-BRC and NCBI GenBank databases, and accession numbers for all strains are listed in the main text and Supplementary Information.

## Supplementary Materials

Supplementary 1 Supplementary Results Figs. S1 to S4 Tables S1 to S8 Dataset S1
