# Supplementary material for: Predicting Strain-Specific Metabolic Capabilities in the Genus Pseudomonas with a Flux-to-AI Approach Reveals Hidden Cell Envelope Properties
Source: Comput Struct Biotechnol J. 2026 Aug 13;35(1):0190. doi: 10.34133/csbj.0190 (PMC13469259; doi:10.34133/csbj.0190)
Supplement: Supplementary file 1 [file csbj.0190.f1.zip › Supplementary Materials Pseudomonas Manuscript.docx]

Supplementary Materials

Predicting strain-specific metabolic capabilities in the Genus Pseudomonas using a Flux-to-AI approach unravels hidden cell envelope properties.

Carlos Focil, Christopher Dalldorf, Diego Martinez, Alejandro Zepeda, Cristal Zuniga

**This PDF file includes:**

Expanded Results

Supplementary Figures 1-3

Captions for Supplementary Tables 1 to 8

Captions for Supplementary Dataset 1

References of the Methods

**Other Supplementary Materials for this manuscript include the following:**

Supplementary Tables 1 to 3

Dataset 1

# **Results**

## **Comparative genomics of Pseudomonas strains**

A total of 44 Pseudomonas genomes, representing different species and strains, were obtained from the PATRIC and NCBI GenBank databases. These genomes encompassed species such as *P. aeruginosa, P. putida, P. fluorescens, P. protegens, P. asiatica, P. campi,* and other *Pseudomonas* spp. (Figure 1A). The most abundant species in the dataset were *Pseudomonas sp., P. aeruginosa, P. putida, and P. fluorescens*. To explore their evolutionary relationships, 16S rRNA sequences were extracted from the GenBank annotations, and a phylogenetic tree was generated using the ape and ggtree libraries from R27,28 (Figure 1B).

**GEM-predicted growth capabilities differentiate strains**

After generating the functional strain-specific models, we assessed their ability to reproduce known *Pseudomonas* phenotypes (Figures 2 and 3). Model validation was performed by simulating both aerobic and anaerobic growth using acetate, glucose, fructose, sucrose, and glutamate as carbon sources. Additionally, the degradation of xenobiotic compounds such as toluene and benzene was simulated, as these compounds have been widely reported to be degraded by *Pseudomonas* species. Under aerobic conditions, *P. fluorescens* and *P. alcaligenes* exhibited the lowest substrate versatility, growing only on glucose, fructose, glycerol, and sucrose, suggesting limited adaptability to environmental nutrient fluctuations. In contrast, *P. aeruginosa, P. putida, P. protegens*, and *P. zhaodongensis* displayed the highest metabolic versatility, successfully utilizing most of the tested carbon sources and demonstrating the ability to degrade toluene and benzene.

**GEMs enable the investigation of species-specific auxotrophies**

To gain further insight into the nutritional preferences of the species, we employed the strain-specific GEMs to identify and characterize potential auxotrophies (see Methods). By systematically deleting external genes from each model, we found that several strains were unable to synthesize at least one biomass component when simulated in M9 minimal medium supplemented with glucose. Consequently, we evaluated the effect of supplementing these models with additional extracellular nutrients under the same conditions. Among the 20 tested nutrients, potential auxotrophies were identified for five compounds: L-isoleucine, L-leucine, L-serine, L-valine, and ornithine (Figure 4). The L-serine auxotrophy exhibited the highest number of associated genes and was most prevalent in *P. zhaodongensis, Pseudomonas sp., P. campi,* and *P. asiatica*. In contrast, auxotrophies for L-isoleucine, L-leucine, L-valine, and ornithine were more commonly observed in *P. putida, P. protegens*, and *P. fluorescens*.

**Catabolic capabilities differentiate *Pseudomonas* species**

Given the previously observed differences in carbon source utilization and auxotrophic profiles among Pseudomonas species and strains, we hypothesized that these traits may reflect functional variations in their ability to thrive under diverse nutritional environments. To explore this, we constructed a classification framework for Pseudomonas species based on their GEM-predicted metabolic capabilities. Specifically, we simulated biomass production across more than 405 nutrient conditions by testing growth on all available carbon sources under both aerobic and anaerobic environments. The resulting growth profiles were then used as features for species classification (Figure 3).

**Comparing metabolic and metabolic expression models**

Overall, the ME models exhibited higher fluxes through amino acid–related pathways than the M models, likely reflecting the increased amino acid demand associated with the additional enzyme and mRNA synthesis requirements of the ME models. Interestingly, despite showing enhanced activity in transport-related genes, the ME models displayed reduced activity in cell envelope pathways. This observation aligns with previous findings indicating that *Pseudomonas* species possess few general porins and instead rely on highly specific import porins, a characteristic that contributes to their remarkable drug and stress tolerance. Such metabolic shifts may underlie functional differences in environmental adaptability and pathogenic potential.

# **Supplemental Figures**

# **Supplementary Figure S1.** Number of genes conserved in draft strain-specific models across three PID threshold values a) 80%, b) 70%, and c) 60%.

**Supplementary Figure S2.** Predicted growth capabilities of the 44 strain-specific *Pseudomonas* GEMs. The simulations were performed in M9 minimal medium (**Supplementary Table 3**) under aerobic conditions. Uptake rates were constrained to 17 and 20 mmol gDW^-1^h^-1^ for the carbon sources and oxygen, respectively. The *Pseudomonas* species and their GenBank accession numbers are in rows. Carbon source tested in columns. Color code indicates 1 = growth, 0 = no growth.

**Supplementary Figure S3.** Predicted growth capabilities of the 44 strain-specific *Pseudomonas* GEMs. The simulations were performed in M9 minimal medium under anaerobic conditions. Uptake rates were constrained to 17 and 20 mmol gDW^-1^h^-1^ for the carbon sources and nitrate, respectively. Oxygen uptake rate was constrained to 0 mmol gDW^-1^h^-1^. The *Pseudomonas* species and their GenBank accession numbers are in rows. Carbon source tested in columns. Color code indicates 1 = growth, 0 = no growth.

**Supplementary Figure S4**. Testing robustness of decision tree classifier using a balanced sample of four *Pseudomonas* species, using the 405 simulated conditions across 44 strain-specific models. **A)** Bar plots represent the number of genomes per strain. **B)** Bar plots represent the classes that are split by the decision tree based on the ability of the models to grow on each substrate. Arrows show the outcome of the decision. Pie charts at the bottom of the decision tree represent the final classifications, showing the most representative class for each. Bar plots show all metabolites broken down on the x-axis of several trees, and on the y-axis the number of times that they appeared, and are sorted by appearances. Bolded metabolites were identified in the 13 *Pseudomonas* analyses.

# **Captions of Supplemental Tables**

**Supplementary Table 1.** Reactions were added as an orphan reaction.

**Supplementary Table 2.** The non-essential external genes were eliminated from the models.

**Supplementary Table 3.** Predicted growth capacities in the minimum composition of M9 medium under anaerobic and aerobic conditions.

**Supplementary Table 4.** Robustness analysis of metabolic capabilities that are not critical for the decision tree.

**Supplementary Table 5.** Biomass objective functions of previous *Pseudomonas* models.

**Supplementary Table 6.** Model properties of reactions and metabolites of *P. aeruginosa* model (LR590473).

**Supplementary Table 7.** Predicted carbon utilization growth phenotypes in *P. aeruginosa*

**Supplementary Table 8.** Predicted flux variability by *Pseudomonas* ME-models

# **Captions of Supplemental Data**

**Supplementary Data 1.** All data downloaded was cleaned and checked for duplicate entries, resulting in 44 unique genomic sequences. The file can be found at <https://github.com/cristalzucsd/Pseudomonas>

**References**

1.	Schellenberger, J. *et al.* Quantitative prediction of cellular metabolism with constraint-based models: the COBRA Toolbox v2.0. *Nat. Protoc.* **6,** 1290–1307 (2011).

2.	Basan, M. *et al.* Overflow metabolism in *Escherichia coli* results from efficient proteome allocation. *Nature* **528,** 99–104 (2015).

3.	Paczia, N. *et al.* Extensive exometabolome analysis reveals extended overflow metabolism in various microorganisms. *Microb. Cell Fact.* **11,** 122 (2012).

4.	Schimek, C. in *Evolution of Fungi and Fungal-Like Organisms* 293–329 (2011).

5.	Zuñiga, C., Zaramela, L. & Zengler, K. Elucidation of complexity and prediction of interactions in microbial communities. *Microb. Biotechnol.* **10,** 1500–1522 (2017).

6.	Cárdenas, J., Zumft, W. G. & Morena, C. G. in *Bioenergetics & metabolism of green algae* 75–82 (1974).

7.	Zuñiga, C. *et al.* Predicting dynamic metabolic demands in the photosynthetic eukaryote *Chlorella vulgaris*. *Plant Physiol.* **176,** 450–462 (2018).

8.	Zuñiga, C. *et al.* Genome-scale metabolic model for the green alga *Chlorella vulgaris* UTEX 395 accurately predicts phenotypes under autotrophic, heterotrophic, and mixotrophic growth conditions. *Plant Physiol.* **172,** 589–602 (2016).

9.	Matthews, B. W. Comparison of the predicted and observed secondary structure of T4 phage lysozyme. *BBA - Protein Struct.* **405,** 442–451 (1975).
